# Predictive Value of Anxiety State During Acute Herpes Zoster for the Development of Postherpetic Neuralgia

**DOI:** 10.62641/aep.v54i3.2214

**Published:** 2026-06-15

**Authors:** Zhe Ling, Mingyang Ren

**Affiliations:** ^1^Department of Dermatology, Haining Central Hospital, 314400 Jiaxing, Zhejiang, China; ^2^Department of Dermatology, BenQ Medical Center, The Affiliated BenQ Hospital of Nanjing Medical University, 210019 Nanjing, Jiangsu, China

**Keywords:** herpes zoster, postherpetic neuralgia, anxiety, risk factor, prediction model, central sensitization

## Abstract

**Background:**

Postherpetic neuralgia (PHN) remains the most frequent and distressing sequela of herpes zoster (HZ). Although psychological factors are known to contribute to chronic pain, their role during the acute phase of HZ in predicting PHN has not been fully clarified. This study aimed to investigate whether acute-phase anxiety is independently associated with subsequent PHN development and to evaluate the added predictive value of anxiety assessment in clinical prediction models.

**Methods:**

This longitudinal cohort study enrolled 99 patients with acute HZ between January 2022 and January 2025. Anxiety was assessed at baseline using the Hospital Anxiety and Depression Scale-Anxiety subscale (HADS-A) and the State-Trait Anxiety Inventory-State subscale (STAI-S). PHN was defined as pain lasting at least 90 days after rash onset. We performed logistic regression to identify independent predictors and constructed receiver operating characteristic (ROC) curves to evaluate predictive performance.

**Results:**

The 3 months incidence of PHN was 41.4% (41/99). Patients who developed PHN had significantly higher baseline HADS-A scores (10.7 ± 3.8 vs. 6.4 ± 3.2, *p* < 0.001) and STAI-S scores (52.8 ± 10.4 vs. 41.3 ± 9.7, *p* < 0.001) than those who did not. Multivariate analysis identified three independent predictors: age (adjusted odds ratio [OR] = 1.47 per 10 years, *p* = 0.038), baseline pain intensity (adjusted OR = 1.41, *p* = 0.012), and HADS-A score (adjusted OR = 1.31 per unit, *p* = 0.001). Clinically significant anxiety (HADS-A ≥ 8) was associated with a 4.52-fold increased risk of PHN (95% confidence interval: 1.72–8.58, *p* = 0.013). HADS-A demonstrated good discriminative ability (area under the curve [AUC] = 0.813), and a combined model incorporating anxiety with clinical variables achieved superior predictive performance (AUC = 0.876 vs. 0.762, *p* = 0.019).

**Conclusions:**

Acute-phase anxiety is an independent and clinically meaningful predictor of PHN. Adding anxiety assessment to clinical evaluation significantly improves risk prediction accuracy, supporting routine psychological screening and early intervention in patients with HZ.

## Introduction

Herpes zoster (HZ), commonly known as shingles, results from reactivation of latent varicella–zoster virus in sensory ganglia after previous varicella infection. Over recent decades, the worldwide incidence of HZ has gradually increased [1]. The lifetime risk is one-quarter to one-third of adults, and risk is substantially higher in older and immunocompromised populations [2]. Clinically, acute HZ presents with unilateral dermatomal vesicular rash, accompanied by various symptoms including insomnia, anxiety, and malaise.

In some patients, pain persists long after rash resolution, a condition defined as postherpetic neuralgia (PHN) [3, 4]. Reported PHN incidence varies widely (5%–30%) depending on definition, population, and follow-up duration [5]. PHN remains challenging to treat; current therapies provide inadequate pain relief in up to 50% of affected patients [6]. Well-established clinical predictors of PHN include advanced age, higher acute pain intensity, more severe rash, presence of prodromal symptoms, and immunocompromised status [7]. Recent meta-analyses have also identified chronic obstructive pulmonary disease, hypertension, malignancy, and psychological conditions such as anxiety and depression as risk factors [8]. Despite these advances, clinical prediction of PHN remains imprecise, and the relationship between acute-phase psychological responses and chronic pain development has not been fully elucidated.

The relationship between psychological factors and chronic pain has received increasing attention, particularly in the context of central sensitization—a state of amplified neural responsiveness in the central nervous system that leads to hyperalgesia and allodynia [9, 10]. Evidence suggests that anxiety contributes to this process through multiple mechanisms: it can activate sympathetic arousal, dysregulate the hypothalamic–pituitary–adrenal (HPA) axis, and promote neuroinflammation, thereby enhancing pain sensitization and impairing endogenous pain modulation [11, 12]. Furthermore, a study has demonstrated that pre-existing anxiety and emotional distress are associated with decreased pain tolerance, increased pain perception, and a higher probability of persistent pain after acute injury or inflammation [13].

In HZ, acute-phase psychological factors may be important determinants of pain chronification. Preliminary study suggested that patients who later develop PHN may have higher levels of anxiety and depressive symptoms during the initial infection [14]. However, evidence on anxiety as an independent prospective predictor of PHN remains inconsistent. Some prospective studies have found no significant association between acute-phase anxiety scores and PHN risk [15, 16], while other study suggests psychological distress predicts poor outcomes [17]. These discrepancies may be attributable to methodological differences, including varied assessment tools, inconsistent PHN definitions, heterogeneous populations, and small sample sizes.

Given the substantial burden of PHN and the potentially modifiable nature of psychological factors, better understanding of the relationship between acute-phase anxiety and pain chronification is urgently needed Therefore, this prospective study was designed to evaluate the predictive role of acute-phase anxiety in PHN development and severity. We prospectively enrolled patients with acute HZ from January 2022 to January 2025, systematically assessed anxiety using standardized instruments, and followed them to determine PHN outcomes. This study aimed to provide evidence for incorporating psychological assessment with clinical variables and to identify potential targets for early intervention.

## Methods

### Study Design

This prospective cohort study was conducted at the Department of Pain Medicine and Dermatology, Haining Central Hospital, from January 2022 to January 2025. The primary objective was to examine whether anxiety symptoms at HZ onset are associated with subsequent PHN. Participants were monitored for at least six months after rash onset to ascertain PHN outcomes. The study protocol was approved by the Haining Central Hospital Institutional Ethics Committee (Approval No.2006001). All procedures adhered to internationally accepted ethical principles for medical research involving human subjects. Written informed consent was obtained from each participant prior to enrolment. The study was conducted in accordance with the Declaration of Helsinki [18].

### Participants

Patients with acute HZ were consecutively recruited from the outpatient clinics and inpatient wards. HZ diagnosis was based on unilateral dermatomal vesicular rash with localized pain or sensory abnormalities. The inclusion criteria were as follows: (1) age ≥18 years; (2) confirmed acute HZ within 14 days of rash onset [6]; (3) no previous HZ episodes; (4) ability to complete study questionnaires; and (5) agreement to attend follow-up assessments. The exclusion criteria were as follows: (1) pre-existing chronic pain conditions in the affected dermatome; (2) severe cognitive impairment or psychiatric disorders precluding reliable assessment; (3) current regular use of psychotropic medication for anxiety or depression (patients completed a brief psychiatric history questionnaire at baseline to record pre-existing anxiety or depression diagnoses); (4) immunocompromised status due to human immunodeficiency virus (HIV), organ transplantation, or active malignancy requiring chemotherapy; (5) pregnancy or lactation; or (6) anticipated inability to complete follow-up due to relocation or terminal illness.

### Sample Size Calculation

Sample size was estimated using previously reported PHN incidence rates of approximately 15–20% among patients with HZ and an anticipated difference in anxiety scores between PHN and non-PHN groups [1]. With a two-sided significance level of 0.05, a statistical power of 80%, and an expected odds ratio (OR) of 2.5 for high anxiety predicting PHN [19], we calculated that at least 80 participants were required. Accounting for an expected attrition rate of approximately 15% during follow-up, we aimed to enrol at least 95 patients. Sample size calculation was performed using PASS software (version 15.0; NCSS, LLC, Kaysville, UT, USA) with the formula: n = [(Z_α/2_ + Z_β_)^2^ × {P_1_(1 – P_1_) + P_2_(1 – P_2_)}] / (P_1_ – P_2_)^2^, where n = required sample size per group, $Z_{\alpha /2}$ = 1.96 for two-sided, α = 0.05. Z_β_ = 0.84 for β = 0.20, β = 0.20 (power = 80%), P_1_ = proportion of PHN in the low-anxiety group (baseline risk), P_2_ = proportion of PHN in the high-anxiety group.

### Data Collection and Assessment Tools

Baseline demographic and clinical information was recorded within 24 hours of enrolment, including age, sex, and comorbidities (e.g., diabetes, hypertension, cardiovascular disease). Disease-specific variables included time from rash onset to presentation, dermatomal location (e.g., cranial, cervical, thoracic, lumbar, or sacral), rash severity graded on a four-level scale according to lesion extent and vesicle density, presence of prodromal symptoms, and antiviral treatment regimen. All patients received oral valacyclovir (1 g, three times daily) for 7 days, with no variation in drug type or dosage. Analgesic treatment followed the World Health Organization pain ladder. paracetamol for mild pain, non-steroidal anti-inflammatory drugs (NSAIDs) for moderate pain, and NSAIDs plus weak opioids for severe pain.

Anxiety was assessed using the Hospital Anxiety and Depression Scale-Anxiety subscale (HADS-A) [20] and the State-Trait Anxiety Inventory-State subscale (STAI-S) [21]. HADS-A consists of seven items scored from 0 to 3, yielding total scores ranging from 0 to 21, with higher values indicating greater anxiety. A score of ≥8 was used to define clinically significant anxiety. 7 items: Item 1: I feel tense or “wound up”; Item 3: I get a sort of frightened feeling as if something awful is about to happen; Item 5: Worrying thoughts go through my mind; Item 7: I can sit at ease and feel relaxed; Item 9: I get a sort of frightened feeling like “butterflies” in the stomach; Item 11: I feel restless as if I have to be on the move; Item 13: I get sudden feelings of panic. Cronbach’s α is 0.81.

The STAI-S comprises 20 items rated on a four-point Likert scale, generating scores between 20 and 80. Both instruments have been validated in Chinese populations and demonstrate good psychometric properties for anxiety assessment in medical settings. The content assesses subjective feelings of tension, apprehension, nervousness, worry, and autonomic nervous system arousal. It comprises a balanced mix of items reflecting anxious emotions and reverse-scored items representing calm states. All items are rated on a 4-point intensity scale from not at all to very much so, with total scores ranging from 20 to 80; higher scores indicate severe state anxiety. Cronbach’s α is 0.84.

Depression was assessed using the Hospital Anxiety and Depression Scale-Depression subscale (HADS-D) [22]. The HADS-D consists of seven items assessing anhedonia and loss of interest, core features of depression, while specifically excluding somatic symptoms that might be confounded with physical illness. The items focus on reduced pleasure, loss of interest in appearance, diminished ability to laugh, and lack of optimism about the future. Additionally, it captures feelings of slowing down and the capacity to enjoy entertainment. All items are rated on a 4-point Likert scale (0–3) based on the patient feeling over the past week. Total scores ranging from 0 to 21, with higher scores indicating greater depressive symptomatology, typically categorized as normal (0–7), mild (8–10), moderate (11–14), or severe (15–21). Cronbach’s α is 0.83.

Pain severity was quantified using an 11-point Numeric Rating Scale (NRS) [23], anchored at 0 (“no pain”) and 10 (“pain as severe as imaginable”). Participants reported their present pain intensity as well as average and maximum pain during the preceding 24 hours.

Qualitative pain characteristics were assessed with the Short-Form McGill Pain Questionnaire (SF-MPQ) [24], which includes sensory and affective descriptors. SF-MPQ consists of three primary components, including the pain rating index: This index includes 15 descriptive adjectives (11 sensory and 4 affective), each rated on a 0–3 intensity scale.

Neuropathic features were evaluated using the Leeds Assessment of Neuropathic Symptoms and Signs (LANSS) [25] was administered to evaluate the neuropathic characteristics of pain. It consists of two main parts: the first is a 5-item patient-reported questionnaire that assesses specific sensory disturbances (e.g., the presence of pins and needles), changes in skin colour or sweating, and sensations of heat or burning. The second part involves a brief clinical examination by a practitioner to detect the presence of allodynia and an altered pinprick threshold. Each of the seven total items is assigned a weighted score, and a total score of 12 or higher (out of a maximum of 24) indicates that neuropathic mechanisms are likely contributing to the patient’s pain. Cronbach’s α is 0.84.

Rash severity was graded as follows: mild, defined as rash involving less than one-fourth of the neve-innervated dermatome; moderate, rash involving one-fourth to three-fourths of the dermatome; and severe/very severe, rash involving more than three-fourths of the dermatome [26].

### Definition of PHN and Outcome Measures

The primary endpoint of the study was PHN, defined as pain persisting in the affected dermatome for at least 90 days after rash onset [27]. Secondary outcomes included: (1) PHN severity classified by average 3-month NRS scores: mild (1–3), moderate (4–6), severe (7–10) [28]; (2) time to pain resolution in non-PHN patients; and (3) pain-related functional interference assessed by the Brief Pain Inventory–Short Form [29].

### Follow-up Procedures

Follow-up assessments were conducted at 1, 3 and 6 months after rash onset. At each visit, patients underwent standardized evaluation of pain status (e.g., current NRS, pain quality, analgesic use). The 3-month assessment served as the primary time point for determining PHN status. Patients unable to attend in-person visits were contacted by telephone. Those who failed to respond to three consecutive contact attempts were classified as lost to follow-up. All patients received routine clinical management (e.g., antiviral and analgesic agents) as clinically indicated; treatment decisions were made independently by attending physicians and were not influenced by study participation.

### Statistical Analysis

Statistical analyses were performed using SPSS (version 26.0; International Business Machines Corporation, Armonk, NY, USA) and R software (version 4.2.0; R Foundation for Statistical Computing, Vienna, Austria). A two-tailed significance threshold of 0.05 was used. Continuous variables were summarized as mean ± standard deviation for normally distributed data or median with interquartile range for non-normal distributions. Categorical variables were expressed as counts and percentages. Between-group comparisons used independent-sample *t*-tests or Mann–Whitney U tests for continuous variables and chi-square or Fisher’s exact tests for categorical variables, as appropriate. Pearson correlation was used to assess the relationship between HADS‑A and HADS-D scores.

To explore determinants of PHN, logistic regression modelling was used to identify relevant predictors. In univariate logistic regression analysis, variables yielding associations with *p* < 0.10 in preliminary analyses, along with clinically relevant covariates, were incorporated into multivariable models. Multicollinearity was assessed using variance inflation factors (VIFs). A conventional threshold of VIF <2.5 was defined as acceptable multicollinearity; variables with VIF ≥2.5 were considered to have significant collinearity and were excluded or adjusted to stabilize regression estimates. Adjusted ORs with corresponding 95% confidence intervals (CIs) were reported. Model fit was evaluated using the Hosmer–Lemeshow test.

Discriminative performance of anxiety measures and combined prediction models was assessed using receiver operating characteristic (ROC) curves. The area under the curve (AUC) quantified predictive accuracy, thresholds of 0.7 to <0.8, 0.8 to <0.9, and >0.9 were considered acceptable, excellent, and outstanding, respectively [30]. Optimal cut-off values were determined using the Youden index. AUC comparisons used the DeLong method. Internal validation of the predictive model was performed using bootstrap resampling with 1000 iterations to generate an optimism adjusted AUC, which was used to evaluate model stability and reduce overfitting bias.

Strict assessor blinding was implemented: the research team was divided into two independent groups: assessment group (baseline anxiety or pain rating and follow-up data collection) and outcome adjudication group (PHN diagnosis based on follow-up pain data). The assessment group was unaware of future PHN outcomes, and the outcome adjudication group had no access to the baseline anxiety scores. All data were de-identified and coded before statistical analysis to eliminate assessor bias, and this blinding protocol is now explicitly described in the manuscript.

## Results

### Patient Enrolment and Follow-up

Between January 2022 and January 2025, 127 patients presenting with acute HZ were screened for eligibility. Of these, 23 were excluded: 8 due to immunocompromised status (3 with active malignancy on chemotherapy, 3 post-transplant, 2 with HIV), 6 due to pre-existing chronic pain, 4 due to cognitive impairment, 3 due to current psychotropic medication use, and 2 due to anticipated inability to complete follow-up. Thus, 104 participants met inclusion criteria and completed baseline evaluations. During follow-up, 5 patients (4.8%) were lost to follow-up: 2 relocated, 2 withdrew consent, and 1 died from unrelated cardiovascular disease. The final analytic cohort comprised 99 patients, exceeding the calculated minimum sample size requirement.

### Baseline Demographic and Clinical Characteristics

Baseline characteristics stratified by anxiety status (HADS-A ≥8 vs. <8; Table 1). Mean age was 58.4 ± 12.7 years (range 24–86), 56.6% were female. Approximately two-thirds were ≥50 years. Thoracic involvement was most common (52.5%), followed by cervical (18.2%), lumbar (15.2%), cranial (11.1%), and sacral (3.0%). Median time from rash onset to clinical presentation was 4 days (Q1–Q3: 2–6). Prodromal symptoms were reported by 61 patients (61.6%). Rash severity was mild in 29.3%, moderate in 37.4%, severe in 33.3%. Mean baseline pain intensity NRS was 5.8 ± 2.1, 72 patients (72.7%) have LANSS ≥12, indicating neuropathic pain. Hypertension (38.4%), diabetes mellitus (22.2%), and cardiovascular disease (15.2%) were common comorbidities. All patients received antiviral therapy within 72 hours of enrolment.

**Table 1.** **Baseline demographic and clinical characteristics by anxiety status**.

Variable		Total (n = 99)	HADS-A ≥8 (n = 41)	HADS-A <8 (n = 58)	χ^2^/t/Z	*p* value
Age (years), mean ± SD		58.4 ± 12.7	62.1 ± 11.8	55.4 ± 12.9	t = 2.58	0.011
Age ≥50 years		67 (67.7%)	31 (75.6%)	36 (62.1%)	χ^2^ = 2.13	0.144
Female		56 (56.6%)	25 (61.0%)	31 (53.4%)	χ^2^ = 0.59	0.442
Median time from rash onset (days)		4 (2, 6)	4 (2, 6)	4 (2, 6)	Z = 1.48	0.071
Rash location					χ^2^ = 2.51	0.644
	Thoracic	52 (52.5%)	21 (51.3%)	31(53.4%)		
	Cervical	18 (18.2%)	10 (24.4%)	8 (13.8%)		
	Lumbar	15 (15.2%)	6 (14.6%)	9 (15.5%)		
	Cranial	11 (11.1%)	3 (7.3%)	8 (13.8%)		
	Sacral	3 (3.0%)	1 (2.4%)	2 (3.4%)		
Comorbidities						
	Hypertension	38 (38.4%)	18 (43.9%)	20 (34.5%)	χ^2^ = 0.05	0.832
	Diabetes mellitus	22 (22.2%)	10 (24.4%)	12 (20.7%)	χ^2^ = 0.19	0.663
	Cardiovascular disease	15 (15.2%)	7 (17.4%)	8 (13.8%)	χ^2^ = 0.20	0.654
Baseline pain (NRS), mean ± SD		5.8 ± 2.1	6.7 ± 1.9	5.1 ± 2.1	t = 3.82	<0.001
Rash severity					χ^2^ = 10.10	0.006
	Mild	32 (32.3%)	10 (24.4%)	22 (37.9%)		
	Moderate	34 (34.3%)	10 (24.4%)	24 (41.4%)		
	Severe/Very severe	33 (33.3%)	21 (51.2%)	12 (20.7%)		
Prodromal symptoms		61 (61.6%)	27 (65.9%)	34 (58.6%)	χ^2^ = 0.56	0.456
Neuropathic pain (LANSS ≥12)		72 (72.7%)	32 (78.0%)	40 (69.0%)	χ^2^ = 1.02	0.312

Note: SD, standard deviation; NRS, Numeric Rating Scale; LANSS, Leeds Assessment of Neuropathic Symptoms and Signs; HADS-A, Hospital Anxiety and Depression Scale-Anxiety subscale.

Patients with clinically significant anxiety were older on average (62.1 ± 11.8 vs. 55.4 ± 12.9 years, *p* = 0.011), but the proportion of patients aged ≥50 years did not differ significantly (75.6% vs. 62.1%, *p* = 0.144)—likely due to the variability in age distribution within the ≥50 years subgroup (e.g., a higher proportion of young-old patients [50–64 years] in the non-anxiety group and a higher proportion of old-old patients [≥65 years] in the anxiety group). This age distribution heterogeneity is the main reason for the non-significant difference in the categorical age variable. Patients in HADS-A ≥8 group also exhibited higher baseline pain intensity (6.7 ± 1.9 vs. 5.1 ± 2.1, *p* < 0.001) and severe or very severe rash (51.2% vs. 24.6%, *p* = 0.006). No other significant differences were observed between anxiety groups with respect to sex distribution, dermatomal location, time to presentation, prodromal symptoms, or comorbidities (Table 1).

### Incidence and Severity of PHN

At 3-month follow-up assessment, 41 of 99 patients (41.4%) met the diagnostic criteria for PHN, and 58 (58.6%) had complete pain resolution. PHN incidence was markedly higher in patient with clinically significant anxiety compared with those without anxiety (63.4% vs. 25.9%, χ^2^ = 14.27, *p* < 0.001). Among the 41 patients with PHN, pain severity was mild (NRS 1–3) in 12 patients (29.3%), moderate (NRS 4–6) in 19 (46.3%), and severe (NRS 7–10) in 10 (24.4%). At six-month, 32 patients (32.3%) had persistent pain, indicating that 9 patients with PHN at 3 months subsequently achieved pain resolution between the third and sixth month (Table 2). Mean time to complete pain resolution in non-PHN patients was 42.3 ± 18.6 days.

**Table 2.** **Incidence and severity of PHN by anxiety status**.

Variable	Total (n = 99)	HADS-A ≥8 (n = 41)	HADS-A <8 (n = 58)	χ^2^/t	*p*
PHN at 3 months, Present	41 (41.4%)	26 (63.4%)	15 (25.9%)	χ^2^ = 14.27	<0.001
PHN severity - Mild (NRS 1–3)	12 (29.3%)	6 (23.1%)	6 (40.0%)	χ^2^ = 1.89	0.389
PHN severity - Moderate (NRS 4–6)	19 (46.3%)	13 (50.0%)	6 (40.0%)		
PHN severity - Severe (NRS 7–10)	10 (24.4%)	7 (26.9%)	3 (20.0%)		
PHN at 6 months, Persistent	32 (32.3%)	21 (51.2%)	11 (19.0%)	χ^2^ = 11.35	0.001

Note: PHN, postherpetic neuralgia; NRS, Numeric Rating Scale; HADS-A, Hospital Anxiety and Depression Scale-Anxiety subscale.

### Comparison of Anxiety Levels Between PHN and Non-PHN Groups

**Fig. 1.**
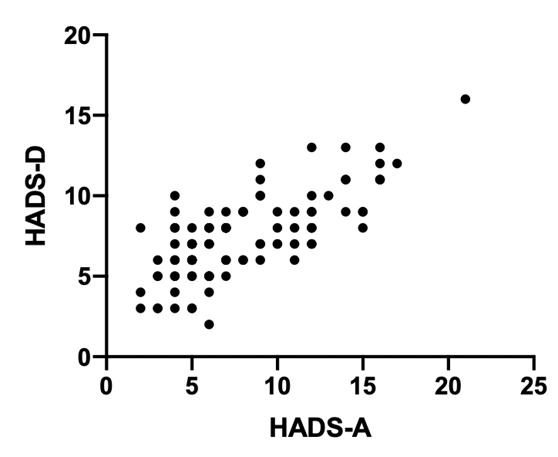
**Correlation between HADS-A and HADS-D scores**. Correlation analysis showed a moderately strong positive correlation between HADS-A and HADS-D scores (Pearson r = 0.58, n = 99, *p* < 0.001). HADS-A, Hospital Anxiety and Depression Scale-Anxiety subscale; HADS-D, Hospital Anxiety and Depression Scale-Depression subscale.

This study enrolled 99 patients with acute HZ, of whom 41 (41.4%) developed PHN at the 3-month follow-up (PHN group), while 58 (58.6%) achieved complete pain resolution (non-PHN group). Baseline anxiety levels differed significantly between PHN and non-PHN groups. PHN patients had higher HADS-A scores (10.7 ± 3.8 vs. 6.4 ± 3.2, t = 5.42, *p* < 0.001), higher STAI-S scores (52.8 ± 10.4 vs. 41.3 ± 9.7, t = 4.89, *p* < 0.001), and HADS-D scores (8.3 ± 3.5 vs. 5.6 ± 2.9, t = 3.67, *p* < 0.001) than in the non-PHN group. Using HADS-A ≥8 to define clinically significant anxiety, 63.4% (26/41) of patients in the PHN group had significant anxiety compared with 25.9% (15/58) in the non-PHN group (χ^2^ = 14.27, *p* < 0.001). Correlation analysis revealed a moderate association between HADS-A and HADS-D scores (r = 0.58, *p* < 0.001, Fig. 1), indicating that anxiety and depression are partially independent constructs (Table 3).

**Table 3.** **Comparison of anxiety levels between PHN and Non-PHN groups**.

Variable	PHN Group (n = 41)	Non-PHN Group (n = 58)	t/χ^2^	*p* value
HADS-A score (mean ± SD)	10.7 ± 3.8	6.4 ± 3.2	t = 5.42	<0.001
STAI-S score (mean ± SD)	52.8 ± 10.4	41.3 ± 9.7	t = 4.89	<0.001
HADS-D score (mean ± SD)	8.3 ± 3.5	5.6 ± 2.9	t = 3.67	<0.001
Clinically significant anxiety (HADS-A ≥8)	26 (63.4%)	15 (25.9%)	χ^2^ = 14.27	<0.001

Note: PHN, postherpetic neuralgia; HADS-A, Hospital Anxiety and Depression Scale-Anxiety subscale; HADS-D, Hospital Anxiety and Depression Scale-Depression subscale; STAI-S, State-Trait Anxiety Inventory-State subscale; SD, standard deviation.

### Univariate Analysis of Risk Factors for PHN Development

To examine factors associated with PHN onset, separate logistic regression models were constructed for each candidate variable (Table 4). Age demonstrated a significant positive association, with each 10-year increase corresponding to an OR of 1.58 (95% CI: 1.18–2.12, *p* = 0.002). Although female sex demonstrated a higher estimated odds of PHN (OR = 1.76, 95% CI: 0.64–4.84, *p* = 0.273). Baseline acute pain intensity was a strong predictor, with an OR of 1.52 per unit increase in NRS score (95% CI: 1.21–1.91, *p* < 0.001). Compared with mild or moderate rash, severe or very severe rash was associated with substantially elevated odds of PHN (OR = 3.42, 95% CI: 1.28–9.14, *p* = 0.014). The presence of prodromal symptoms conferred an OR of 2.67 (95% CI: 0.91–7.83, *p* = 0.074). Among comorbidities, diabetes mellitus was significantly associated with PHN development (OR = 2.89, 95% CI: 1.04–8.03, *p* = 0.042), while hypertension showed a non-significant trend (OR = 2.21, 95% CI: 0.84–5.82, *p* = 0.108). Most notably, both anxiety measures were strongly associated with PHN: HADS-A (OR = 1.38 per unit, 95% CI: 1.19–1.60, *p* < 0.001). Similarly, STAI-S scores demonstrated a significant incremental effect (OR = 1.09 per point, 95% CI: 1.04–1.14, *p* < 0.001). In categorical anxiety analysis, participants with clinically significant anxiety (HADS-A ≥8) had markedly higher odds of PHN (OR = 6.78, 95% CI: 2.32–19.82, *p* < 0.001).

**Table 4.** **Univariate analysis of risk factors for PHN development**.

Variable	Category/Unit	OR	95% CI	*p*
Age	Per 10-year increment	1.58	1.18–2.12	0.002
Sex	Female vs. Male	1.76	0.64–4.84	0.273
Acute pain intensity (NRS)	Per unit increase	1.52	1.21–1.91	<0.001
Rash severity	Severe/Very severe vs. Mild/Moderate	3.42	1.28–9.14	0.014
Prodromal symptoms	Present vs. Absent	2.67	0.91–7.83	0.074
Diabetes mellitus	Present vs. Absent	2.89	1.04–8.03	0.042
Hypertension	Present vs. Absent	2.21	0.84–5.82	0.108
HADS-A score	Per unit increase	1.38	1.19–1.60	<0.001
STAI-S score	Per unit increase	1.09	1.04–1.14	<0.001
Clinically significant anxiety	HADS-A ≥8 vs. <8	6.78	2.32–19.82	<0.001

Note: OR, odds ratio; CI, confidence interval; NRS, Numeric Rating Scale; HADS-A, Hospital Anxiety and Depression Scale-Anxiety subscale; STAI-S, State-Trait Anxiety Inventory-State subscale; PHN, postherpetic neuralgia.

### Multivariate Analysis and Independent Predictors of PHN

Multivariable logistic regression identified independent predictors (Table 5). All models were adjusted for the same set of covariates. Model 1 (including age, baseline pain intensity, rash severity, prodromal symptoms, diabetes mellitus, LANSS score, and continuous HADS-A), showed that age (adjusted OR = 1.47 per 10 years, 95% CI: 1.02–2.11, *p* = 0.038), baseline pain intensity (adjusted OR = 1.41, 95% CI: 1.08–1.84, *p* = 0.012), and HADS-A score (adjusted OR = 1.31 per unit, 95% CI: 1.11–1.54, *p* = 0.001) were independent predictors. Model 2 (using categorical HADS-A ≥8 vs. <8) found that clinically significant anxiety remained an independent predictor (adjusted OR = 4.52, 95% CI: 1.72–8.58, *p* = 0.013). Model 3 (using STAI-S instead of HADS-A) showed that STAI-S score was also independent predictive value (adjusted OR = 1.07 per unit, 95% CI: 1.02–1.12, *p* = 0.008). Model 4 (including HADS-A and HADS-D) showed that anxiety retained significance (*p* = 0.006) while depression did not (*p* = 0.284), suggesting that the predictive effect is primarily attributable to anxiety rather than general psychological distress. Model diagnostics for Model 1 were satisfactory: Hosmer–Lemeshow goodness-of-fit test was non-significant (χ^2^ = 6.83, *p* = 0.556), all VIFs for all included predictors were <2.5, excluding significant multicollinearity.

**Table 5.** **Multivariate analysis and independent predictors of PHN**.

Model/Variable		Category/Unit	Adj. OR	95% CI	*p*
Model 1 (HADS-A continuous)					
	Age	Per 10-year increment	1.47	1.02–2.11	0.038
	Baseline pain intensity	Per unit increase	1.41	1.08–1.84	0.012
	HADS-A score	Per unit increase	1.31	1.11–1.54	0.001
Model 2 (HADS-A categorical)					
	Clinically significant anxiety	HADS-A ≥8 vs <8	4.52	1.72–8.58	0.013
Model 3 (STAI-S)					
	STAI-S score	Per unit increase	1.07	1.02–1.12	0.008
Model 4 (HADS-A and HADS-D)					
	HADS-A score	Per unit increase	1.28	1.07–1.53	0.006
	HADS-D score	Per unit increase	1.09	0.93–1.28	0.284

Note: Adj. OR, adjusted odds ratio; CI, confidence interval; HADS-A, Hospital Anxiety and Depression Scale-Anxiety subscale; HADS-D, Hospital Anxiety and Depression Scale-Depression subscale; STAI-S, State-Trait Anxiety Inventory-State subscale; PHN, postherpetic neuralgia. Hosmer–Lemeshow test: χ^2^ =6.83, *p* = 0.556. Variance inflation factor (VIF) < 2.5 for all predictors. Model 1 (HADS‑A continuous): Adjusted for age, baseline pain intensity (Numeric Rating Scale [NRS]), rash severity, prodromal symptoms, diabetes mellitus, Leeds Assessment of Neuropathic Symptoms and Signs (LANSS) score, and continuous HADS‑A score. Model 2 (HADS‑A categorical): Adjusted for the same variables as Model 1, with HADS‑A replaced by categorical anxiety status (HADS‑A ≥8 vs. <8). Model 3 (STAI‑S): Adjusted for the same variables as Model 1, with HADS‑A replaced by continuous STAI‑S score. Model 4 (HADS‑A and HADS‑D): Adjusted for the same variables as Model 1, with additional inclusion of continuous HADS‑D score.

### Predictive Performance of Anxiety for PHN Development

**Fig. 2.**
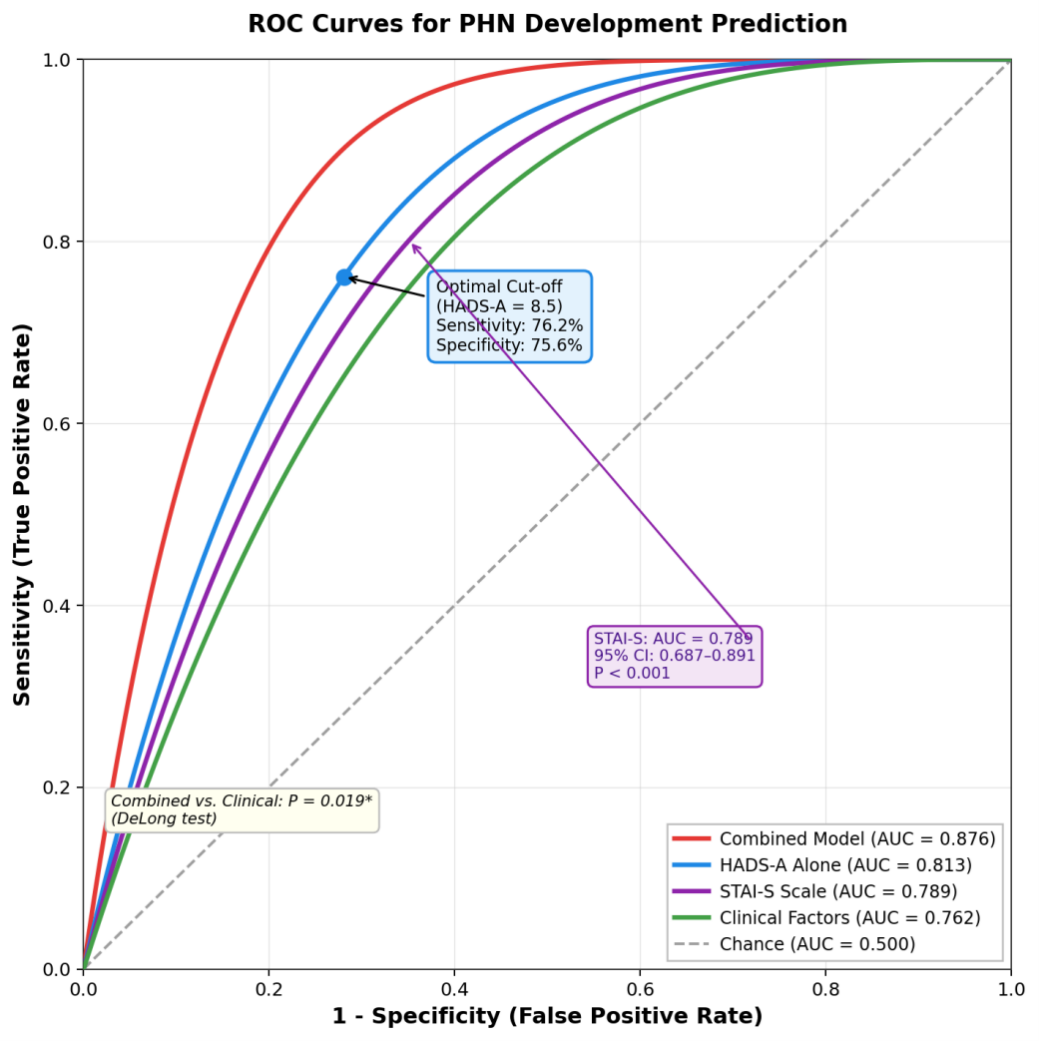
**ROC curves for PHN development prediction**. HADS-A, Hospital Anxiety and Depression Scale-Anxiety subscale; STAI-S, State-Trait Anxiety Inventory-State subscale; ROC, receiver operating characteristic; AUC, area under the curve; PHN, postherpetic neuralgia. Fig. 2 presents the ROC curves comparing the discriminatory performance of different predictive models: HADS-A alone, STAI-S scale, clinical factors only (age, baseline pain intensity, rash severity, prodromal symptoms, diabetes mellitus, and Leeds Assessment of Neuropathic Symptoms and Signs [LANSS] score), and the combined model incorporating both anxiety and clinical factors. The figure clearly demonstrates the superior predictive performance of the combined model (AUC = 0.876) compared to either HADS-A alone (AUC = 0.813) or clinical factors alone (AUC = 0.762).

ROC analysis showed that HADS-A had good discriminative ability (AUC = 0.813, 95% CI: 0.718–0.908, *p* < 0.001). The optimal cut-off (Youden index) was 8.5, with sensitivity 76.2% and specificity 75.6%, positive predictive value 45.7% and negative predictive value 92.2%. STAI-S showed similar performance (AUC = 0.789, 95% CI: 0.687–0.891, *p* < 0.001, optimal cut-off 47.5, sensitivity 71.4%, specificity 73.1%). When anxiety measures were combined with established clinical predictors (age and baseline pain intensity) to build a composite mode based on the clinical factors model, HADS-A score was incorporated to construct the combined model (AUC = 0.876, 95% CI: 0.800–0.952), representing a significant improvement over the clinical-only model (AUC = 0.762), as verified by DeLong comparison testing (Z = 2.34, *p* = 0.019). Internal validation using bootstrap resampling (1000 iterations) produced an optimism-adjusted AUC of 0.854 (95% CI: 0.826–0.882), indicating robust predictive performance. These findings demonstrate that incorporating anxiety assessment significantly enhances the accuracy of PHN risk prediction beyond traditional clinical factors alone (Table 6, Fig. 2).

**Table 6.** **Predictive performance of anxiety for PHN development**.

Analysis/Variable		Value	95% CI	Sens/Spec	*p* value
ROC curve analysis					
	HADS-A score (AUC)	0.813	0.718–0.908	76.2%/75.6%	<0.001
	HADS-A optimal cut-off	8.5	PPV: 45.7%	NPV: 92.2%	
	STAI-S score (AUC)	0.789	0.687–0.891	71.4%/73.1%	<0.001
	STAI-S score optimal cut-off	47.5	PPV: 42.3%	NPV: 90.1%	
Model comparison					
	Clinical-only model (AUC)	0.762	0.693–0.831		
	Combined model (AUC)	0.876	0.800–0.952		
	DeLong test (Combined vs Clinical)	Z = 2.34			0.019
Internal validation					
	Bootstrap resampling (1000 iter.)				
	Optimism-corrected AUC	0.854	0.826–0.882		<0.001

Note: ROC, receiver operating characteristic; AUC, area under the curve; CI, confidence interval; PPV, positive predictive value; NPV, negative predictive value; Sens, sensitivity; Spec, specificity; HADS-A, Hospital Anxiety and Depression Scale-Anxiety subscale; STAI-S, State-Trait Anxiety Inventory-State subscale; PHN, postherpetic neuralgia. Combined model includes age, baseline pain intensity (Numeric Rating Scale [NRS]), and HADS-A score.

## Discussion

In this longitudinal cohort, anxiety symptoms measured at HZ onset were strongly associated with subsequent PHN development, even after adjusting for established clinical risk factors. Patients with clinically pronounced anxiety at baseline had a substantially elevated risk of PHN, and adding anxiety assessment to clinical prediction models significantly improved prognostic accuracy beyond traditional clinical factors alone. These findings underscore the clinical relevance of integrating psychological assessment into routine HZ care and support early, targeted intervention.

The observed association between acute-phase anxiety and PHN development can be understood within the framework of central sensitization, a pathophysiological process increasingly recognized as fundamental to the transition from acute to chronic pain [31]. Central sensitization refers to increased responsiveness and lowered activation thresholds, leading to exaggerated pain signalling even with mild peripheral input [32]. Clinically, this manifests as hyperalgesia and allodynia, which are characteristic features of PHN.

Anxiety modulates pain processing through multiple interconnected mechanisms. Firstly, anxiety is associated with HPA axis dysregulation, leading to sustained cortisol elevation and other stress hormones [33]. While acute cortisol release is adaptive, chronic HPA axis activation promotes neuroinflammation and impairs brain regions involved in pain modulation (e.g., prefrontal cortex, anterior cingulate cortex and hippocampus) [34]. These alterations compromise descending inhibitory pain pathways that normally suppress nociceptive transmission at the spinal level, thereby facilitating central sensitization. However, the mechanistic link between acute-phase state anxiety and central sensitization in PHN requires further validation through basic science research.

Neuroinflammatory processes further provide a plausible mechanistic bridge between psychological distress and chronic pain maintenance [35]. Glial cells, particularly microglia and astrocytes in the spinal cord and supraspinal structures, become activated in response to peripheral nerve injury and psychological stressors. Animal study have shown that activated microglia and astrocytes release pro-inflammatory cytokines—such as interleukin-1β, interleukin-6, and tumour necrosis factor-α that enhance synaptic transmission and promote neuronal hyperexcitability [36]. Although we did not measure inflammatory factors in this study, the role of neuroinflammation in modulating psychological distress and PHN required further mechanistic investigation.

Our findings align with a multidimensional pain model integrating biological injury with cognitive and emotional influences. While the biological substrate of PHN—varicella-zoster virus-induced damage to sensory neurons—is well characterized, the model emphasizes that tissue pathology alone does not explain the marked heterogeneity in clinical outcomes among patients with HZ. The HADS-A cut-off of ≥8 for clinically significant anxiety is a clinically validated threshold widely used in medical populations. The optimal cut-off of 8.5 identified by ROC analysis is a statistical threshold for PHN prediction, which maximizes sensitivity and specificity but less practical for clinical use. We emphasize that both thresholds are clinically meaningful: HADS-A ≥8 is recommended for routine clinical screening of high-risk patients with HZ, while HADS-A = 8.5 can be used for research purposes to optimize PHN risk prediction.

Prior investigations of psychological determinants of PHN have produced heterogeneous findings. The ORs/hazard ratios for PHN associated with psychological factors have varied widely, mainly due to differences in design, sample size, PHN definition, assessment tools for anxiety/depression, follow-up duration, and confounder adjustment [8]. An early retrospective study suggested that patients who later developed PHN had greater psychological distress or social stressors at disease onset [37]. A small prospective study of 19 patients with HZ reported that those who developed PHN had greater depression, anxiety, and disease conviction during the acute phase compared with those who recovered without chronic pain [38]. Previous study has suggested a link between acute-phase psychological distress and PHN; the key contributions of this study are confirming that acute-phase state anxiety is an independent predictor of PHN after rigorous adjustment for established clinical risk factors and depression [17]. We also quantified the incremental predictive value of anxiety assessment for PHN, and developed a validated combined prediction model incorporating anxiety and clinical factors with high discriminatory ability.

Identifying acute-phase anxiety as an independent predictor of PHN has substantial implications for clinical practice. Routine psychological screening of patients with HZ using validated instruments such as the HADS could facilitate early identification of individuals at elevated risk for chronic pain. The HADS-A scale is a brief 7-item questionnaire suitable for routine clinical screening. Such screening incurs almost no additional cost and can identify high-risk patients for targeted intervention, potentially reducing PHN incidence and long-term medical costs of chronic pain management.

Beyond risk stratification, our findings suggest that alleviating anxiety is significantly associated with reduced PHN risk. For patients with HZ who have clinically significant state anxiety (HADS-A ≥8), brief interventions focusing on pain education, relaxation techniques, and activity pacing could be delivered by trained nurses or through digital health platforms. A major strength of this study is its prospective cohort design, which establishes the temporal sequence of anxiety during acute HZ and subsequent PHN development, supporting valid predictive inference. Furthermore, multivariable logistic regression rigorously adjusted for established clinical risk factors, confirming that acute‑phase anxiety has an independent predictive effect on PHN. These methodological features improve the reliability and interpretability of our findings.

### Limitations

Several limitations of this study should be acknowledged. Firstly, the single-centre design and modest sample size may limit external validity. Large-scale multicentre prospective cohort studies are needed to validate the predictive value of acute-phase anxiety for PHN and to refine the prediction model in diverse populations. Secondly, the overlap between anxiety and depressive symptoms makes complete dissection of their independent effects challenging. Future large-sample studies should further explore the independent and interactive effects of anxiety and depression on PHN development. Thirdly, we did not account for potential confounding by pre-clinical PHN-related neural changes, such as early viral-induced peripheral nerve damage and central sensitization, which may induce acute anxiety in patients with HZ rather than anxiety directly promoting PHN development. Therefore, targeted anti-anxiety interventions in acute HZ to prevent PHN are needed to confirm the causality. Importantly, only 41 PHN events were observed in our cohort. According to the events per variable (EPV) principle in epidemiological research, where EPV ≥10 is generally recommended for model stability, a multivariate model could robustly incorporate approximately 4 predictors given the limited number of events. However, the number of variables included in our regression models exceeded this recommended threshold, introducing a risk of model overfitting, which potentially lead to unstable or overestimated effect sizes. Therefore, the independent risk factors identified in this study and effect estimates should be interpreted with caution, and their generalizability requires further validation.

## Conclusions

In conclusion, this prospective study provides evidence that anxiety state during the acute phase of HZ is an independent predictor of PHN development. Adding anxiety assessment to clinical evaluation improves risk prediction accuracy beyond traditional clinical factors alone. These findings support the biopsychosocial model of pain and highlight the potential value of early psychological screening and intervention in patients with acute HZ.

## Availability of Data and Materials

The datasets used and/or analysed during the current study were available from the corresponding author on reasonable request.

## References

[1] Patil A, Goldust M, Wollina U (2022). Herpes zoster: a review of clinical manifestations and management. *Viruses*.

[2] Giannelos N, Curran D, Nguyen C, Kagia C, Vroom N, Vroling H (2024). The incidence of herpes zoster complications: a systematic literature review. *Infectious diseases and therapy*.

[3] Li HL, Gong G, Wang JL, Li FB (2023). Analysis of risk factors for postherpetic neuralgia in patients with postmalignancy herpes zoster. *Pain physician*.

[4] Adriaansen EJM, Jacobs JG, Vernooij LM, van Wijck AJM, Cohen SP, Huygen FJPM (2025). 8. Herpes zoster and post herpetic neuralgia. *Pain practice : the official journal of World Institute of Pain*.

[5] Ding S, Wen S, Kang H, Zhang H, Guo H, Li Y (2024). Association of the incidence of postherpetic neuralgia with early treatment intervention of herpes zoster and patient baseline characteristics: a systematic review and meta-analysis of cohort studies. *International journal of infectious diseases : IJID : official publication of the International Society for Infectious Diseases*.

[6] Kim J, Kim MK, Choi GJ, Shin HY, Kim BG, Kang H (2021). Pharmacological and non-pharmacological strategies for. preventing postherpetic neuralgia: a systematic review and network meta-analysis. *The Korean journal of pain*.

[7] Giusti EM, Lacerenza M, Manzoni GM, Castelnuovo G (2021). Psychological and psychosocial predictors of chronic postsurgical pain: a systematic review and meta-analysis. *Pain*.

[8] Liang B, Wan Y, Su X, Pan X, Zhang Z, Guo X (2025). New Findings on Risk Factors for Postherpetic Neuralgia From 2014 to 2024: A Systematic Review and Meta-Analysis. *Journal of pain research*.

[9] Lu CC, Lin CY, Lu YY, Tsai HP, Lin CL, Wu CH (2024). CDDO regulates central and peripheral sensitization to attenuate post-herpetic neuralgia by targeting TRPV1/PKC-δ/p-Akt signals. *Journal of cellular and molecular medicine*.

[10] Sachau J, Kersebaum D, Hüllemann P, Adolf D, Kabelitz M, Keller T (2023). The association of self-reported symptoms of central sensitization and sleep disturbances in neuropathic pain. *Pain reports*.

[11] Ahn RS, Park JW, Park IS, Shin HJ, Ryu JH, Oh AY (2023). The Involvement of the Hypothalamus-Pituitary-Adrenal Axis in the Development of Hyperalgesia during the Early Postoperative Period. *Neuroendocrinology*.

[12] Sanabria-Mazo JP, Colomer-Carbonell A, Carmona-Cervelló M, Feliu-Soler A, Borràs X, Grasa M (2022). Immune-inflammatory and hypothalamic-pituitary-adrenal axis biomarkers are altered in patients with non-specific low back pain: a systematic review. *Frontiers in immunology*.

[13] Tidmarsh LV, Harrison R, Wilkinson H, Harrington M, Ravindran D, Norwood S (2026). The persistence of psychological distress while waiting for pain management. *British journal of pain*.

[14] Chen R, Wang D, Chen Z, Li J, Zhang C, Xu C (2024). Comprehensive nursing care improves symptoms and quality of life in elderly patients with postherpetic neuralgia. *Scientific reports*.

[15] Du J, Sun G, Ma H, Xiang P, Guo Y, Deng Y (2021). Prevalence and risk factors of anxiety and depression in patients with postherpetic neuralgia: a retrospective study. *Dermatology (Basel, Switzerland)*.

[16] Tang Q, Yuan M, Huang Y, Feng J, Dai X, Fan X (2026). Analysis of self-management behaviors and related factors in patients with postherpetic neuralgia: A multidimensional methodological study. *Geriatric nursing (New York, N.Y.)*.

[17] Wu J, Yang ZL (2021). A qualitative study of the psychological processes in patients with post-herpetic neuralgia. *Frontiers of Nursing*.

[18] World Medical Association (2025). World Medical Association Declaration of Helsinki: Ethical Principles for Medical Research Involving Human Participants. *JAMA*.

[19] Forbes HJ, Thomas SL, Smeeth L, Clayton T, Farmer R, Bhaskaran K (2016). A systematic review and meta-analysis of risk factors for postherpetic neuralgia. *Pain*.

[20] Bjelland I, Dahl AA, Haug TT, Neckelmann D (2002). The validity of the Hospital Anxiety and Depression Scale: an updated literature review. *Journal of psychosomatic research*.

[21] Zingano BL, Guarnieri R, Diaz AP, Schwarzbold ML, Wolf P, Lin K (2019). Hospital Anxiety and Depression Scale-Anxiety subscale (HADS-A) and The State-Trait Anxiety Inventory (STAI) accuracy for anxiety disorders detection in drug-resistant mesial temporal lobe epilepsy patients. *Journal of affective disorders*.

[22] Montazeri A, Vahdaninia M, Ebrahimi M, Jarvandi S (2003). The Hospital Anxiety and Depression Scale (HADS): translation and validation study of the Iranian version. *Health and quality of life outcomes*.

[23] Chauny JM, Paquet J, Lavigne G, Marquis M, Daoust R (2016). Evaluating acute pain intensity relief: challenges when using an 11-point numerical rating scale. *Pain*.

[24] Velez JC, Friedman LE, Barbosa C, Castillo J, Juvinao-Quintero DL, Williams MA (2022). Evaluating the performance of the Pain Interference Index and the Short Form McGill Pain Questionnaire among Chilean injured working adults. *PloS one*.

[25] Bennett M (2001). The LANSS Pain Scale: the Leeds assessment of neuropathic symptoms and signs. *Pain*.

[26] Nagasako EM, Johnson RW, Griffin DR, Dworkin RH (2002). Rash severity in herpes zoster: correlates and relationship to postherpetic neuralgia. *Journal of the American Academy of Dermatology*.

[27] Kim JH, Lee CS, Han WK, Sim JB, Nahm FS (2022). Determining the definitive time criterion for postherpetic neuralgia using infrared thermographic imaging. *Pain and therapy*.

[28] Fujiwara A, Watanabe K, Yoshimura K, Yamamura Y, Ida M, Kawaguchi M (2023). Correlation between pain catastrophizing in acute herpes zoster and postherpetic neuralgia: a retrospective analysis. *Journal of anesthesia*.

[29] Im DD, Jambaulikar GD, Kikut A, Gale J, Weiner SG (2020). Brief pain inventory–short form: a new method for assessing pain in the emergency department. *Pain medicine (Malden, Mass.)*.

[30] Steyerberg EW, Vergouwe Y (2014). Towards better clinical prediction models: seven steps for development and an ABCD for validation. *European heart journal*.

[31] Schlereth T, Heiland A, Breimhorst M, Féchir M, Kern U, Magerl W (2015). Association between pain, central sensitization and anxiety in postherpetic neuralgia. *European journal of pain (London, England)*.

[32] Curatolo M (2024). Central Sensitization and Pain: Pathophysiologic and Clinical Insights. *Current neuropharmacology*.

[33] Hinds JA, Sanchez ER (2022). The role of the hypothalamus-pituitary-adrenal (HPA) axis in test-induced anxiety: assessments, physiological responses, and molecular details. *Stresses*.

[34] Lei AA, Phang VWX, Lee YZ, Kow ASF, Tham CL, Ho YC (2025). Chronic stress-associated depressive disorders: the impact of HPA axis dysregulation and neuroinflammation on the hippocampus-a mini review. *International journal of molecular sciences*.

[35] Campos ACP, Antunes GF, Matsumoto M, Pagano RL, Martinez RCR (2020). Neuroinflammation, pain and depression: an overview of the main findings. *Frontiers in psychology*.

[36] Ji NN, Li ZY, Cao S, Pei B, Jin CY, Li YF (2024). Neuroinflammation in the paraventricular nucleus of the hypothalamus precipitates visceral pain induced by pancreatic cancer in mice. *Journal of gastrointestinal oncology*.

[37] Qiu L, Chen X, Fu J, Chen X, Wang X (2024). Intravenous patient-controlled analgesia with esketamine improves early depressive symptoms in patients with postherpetic neuralgia: a single-center retrospective cohort study. *BMC psychiatry*.

[38] Purwaningtyas NH, Rakhmani AN, Ashomadoni FH (2025). Navigating Polymorbidity in Geriatric Primary Care: A Case of Herpes Zoster and Psychological Stress. *Jurnal Ilmu Kedokteran Keluarga*.

